# Activation of calcium-sensing receptors is associated with apoptosis in a model of simulated cardiomyocytes ischemia/reperfusion^☆^

**DOI:** 10.1016/S1674-8301(10)60042-5

**Published:** 2010-07

**Authors:** Ling Yan, Tiebing Zhu, Tingting Sun, Liansheng Wang, Shiyang Pan, Zhengxian Tao, Zhijian Yang, Kejiang Cao

**Affiliations:** a Department of Cardiology, the First Affiliated Hospital of Nanjing Medical University, Nanjing 210029, Jiangsu Province, China; b Department of Clinical Key Laboratory, the First Affiliated Hospital of Nanjing Medical University, Nanjing 210029, Jiangsu Province, China; c Department of Biochemistry, Nanjing Medical University, Nanjing 210029, Jiangsu Province, China

**Keywords:** calcium sensing receptors, apoptosis, cardiomyocyte, ischemia/reperfusion

## Abstract

**Objective:**

Calcium-sensing receptors (CaSRs) are G-protein coupled receptors which maintain systemic calcium homeostasis and participate in hormone secretion, activation of ion channels, cell apoptosis, proliferation, and differentiation. Previous studies have shown that CaSRs induce apoptosis in isolated adult rat heart and in normal neonatal rat cardiomyocytes by G-protein-PLC-IP_3_ signaling transduction. However, little knowledge is presently available concerning the role of CaSRs in the apoptosis induced by ischemia and reperfusion in neonatal cardiomyocytes.

**Methods:**

Primary neonatal rat ventricular cardiomyocytes were incubated in ischemiamimetic solution for 2 h, and then re-incubated in normal culture medium for 24 h to establish a model of simulated ischemia/reperfusion (I/R). Cardiomyocyte apoptosis was detected by terminal deoxynucleotidyl transferase-mediated dUTP nick end labeling (TUNEL). The expression of CaSRs mRNA was detected by real-time reverse transcription polymerase chain reaction (RT-PCR). In addition, the expressions of caspase-3 and Bcl-2 were analyzed by western blot.

**Results:**

The simulated I/R enhanced the expression of CaSRs and cardiomyocyte apoptosis. GdCl_3_, a specific activator of CaSRs, further increased the expression of CaSRs and cardiomyocyte apoptosis, along with up-regulation of caspase-3 and down-regulation of Bcl-2.

**Conclusion:**

CaSRs are associated with I/R injury and apoptosis in neonatal rat ventricular cardiomyocytes *via* suppressing Bcl-2 and promoting caspase-3 expression.

## INTRODUCTION

The calcium-sensing receptor (CaSR) belongs to family C II of the superfamily of seven-transmembrane (7TM) receptors, also termed G protein-coupled receptors[1], and was first cloned in 1993 from bovine parathyroid gland[2]. Shortly afterwards, it was found in kidney[3], bone[4] and gastrointestinal tract tissues[5]. It has three structural domains: ① an unusually large extracellular domain, characteristic of the family C 7TM receptors; ② a transmembrane domain; ③ an intracellular domain, which is the hydrophilic COOH terminus of the protein[6].

Small increases in extracelluar Ca^2+^ concentration can cause the synthesis of nitric oxide (NO) by vascular endothelium, thus resulting in vessel dilation[7]. Extracelluar Ca^2+^ affects the vascular endothelium response through a G-protein-coupled CaSR. Human CaSR genetic mutations lead to two different types of disease. Inactivating CaSR mutations develop hypocalciuric hypercalcemia and neonatal hyperparathyroidism, while activating CaSR gene mutations are associated with autosomal dominant hypocalcemia with Bartter syndrome type V[8]. CaSR has been regarded as a potential therapeutic target for treatment of these diseases and others such as osteoporosis.

The most important physiological function of CaSR is to regulate systemic calcium homeostasis. Besides extracelluar Ca^2+^, many ligands such as divalent and trivalent cations, L-amino acids, and polyamines can also regulate CaSR activation[9]. Activation of the CaSR elicits complex intracellular signals through modulation of a wide range of intracellular signaling proteins, including G proteins and phospholipase C (PLC), which in turn stimulate inositol triphosphate production, and thereby increase intracellular Ca^2+^ release. Downstream of or in parallel with PLC, the CaSR also activates mitogen-activated protein kinases (MAPKs) and phosphatidylinositol-4-kinase (PI4K). It activates the PKC, ERK1/2, p38 MAPK and JNK pathways which are known to play an important role in extracelluar signaling transmission from the cell surface to nucleus and in cell cycle regulation[10].

In addition to regulating extracellular calcium homeostasis, CaSR is also involved in proliferation, differentiation, apoptosis, gene expression and hormonal secretion[11]. Wang *et al*[12] first demonstrated the existence of CaSR in adult rat ventricular cardiomyocytes. Later, Tfelt-Hansen *et al*[13] showed that CaSR was also expressed in rat neonatal cardiomyocytes. In the cardiovascular system, a functional CasR has been shown to be present in the heart as well as in blood vessels[14]–[17]. Recently investigation by Sun *et al*[18] showed that CaSR can induce apoptosis in normal rat neonatal cardiomyocytes. However, little is known about the role of CaSR in neonatal cardiac apoptosis induced by ischemia and reperfusion. Therefore, in the present study, we established a model of simulated ischemia/reperfusion (I/R) to allow us to investigate the role of CaSR in I/R-induced apoptosis while analyzing the expression of the apoptotic signaling proteins caspase-3 and Bcl-2 in relation to CaSR.

## MATERIALS AND METHODS

The study was approved by the Institutional Animal Research Committee and all animals received humane care in compliance with the Guide for Care and Use of Laboratory Animals published by the National Institutes of Health (NIH Publication 86-23, revised 1986).

### Animal and reagents

Neonatal Wistar rats were purchased from Animal Research Institute of Nanjing Medical University, China. A specific activator of Case (GdCl_3_), a Na^+^/Ca^2+^ exchanger inhibitor (NiCl_2_) and an L-type calcium channel blocker (CdCl_2_) were purchased from Sigma-Aldrich Chemical Co., USA. Reverse-transcriptase and PCR primers were purchased from Shanghai Sino-American Biochemistry Co., China.

### Cell culture

Culture of primary cell from neonatal rat ventricular cardiomyocytes was prepared by the method described previously[19]. Briefly, neonatal ventricular myocytes were prepared from 2- to 3-day-old neonatal Wistar rats (Animal Research Institute of Nanjing Medical University, China). The rats were anesthetized, sacrificed and then immersed in 70% (v/v) ethanol. The ventricles were aseptically removed, washed three times in Hank's solution, and then minced and incubated with 0.25% (w/v) trypsin in Hank's solution for 10 min at 37°C. Addition of an equal volume of RPMI-1640 containing 10% (v/v) fetal bovine serum was used to terminate the digestion. The supernatant was discarded. Then, cells were incubated with fresh trypsin solution for 20 min at 37°C, and the supernatant was collected. The latter digestion step was repeated four times. Cells in the supernatant were isolated by centrifugation for 10 min at 200 *g* at room temperature in a bench-top centrifuge. Cells were resuspended in RPMI-1640 and incubated at 37°C in a humidified atmosphere containing 5% (v/v) CO_2_.

Three days after the cells were seeded, the cultured cardiomyocytes were randomly divided into four groups: ① the control group in which cardiomyocytes were continuously cultured for 26 h in DMEM; ② the simulated I/R group, in which after 2 h ischemia treatment, the cardiomyocytes were treated with reperfusion for 24 h in DMEM; ③ the GdCl_3_ group, in which 300 µmol/L GdCl_3_ was administered to the culture medium at the beginning of reperfusion; ④ the GdCl_3_ + NiCl_2_ + CdCl_2_ group, in which 300 µmol/L GdCl_3_ + 0.2 mmol/L CdCl_2_ + 10 mmol/L NiCl_2_ were added to the culture medium at the start of reperfusion. For controls, equivalent volumes of medium were added. Only cultures consisting of >95% actin-positive cells, determined by counting 300 cells in three different fields, were subjected to analysis.

### A model of simulated ischemia/reperfusion

The experimental protocol used to simulate I/R was a modified version of the method described by Han *et al*[20]. Briefly, the serum-containing incubation medium was replaced with serum-free DMEM before the start of the experiment. The cells then were treated with an ischemic buffer solution (1 mmol/L NaH_2_PO_4_, 24 mmol/L NaHCO_3_, 2.5 mmol/L CaCl_2_, 118 mmol/L NaCl, 16 mmol/L KCl, 0.5 mmol/L sodium EDTA, 20 mmol/L sodium lactate, pH 6.8, 37°C). When pregasing with 95% N_2_ and 5% CO_2_ for at least 5 min, the ischemic buffer was added to the cells, which were then placed in a sealed chamber containing deoxygenation reagent, which caused the consumption of O_2_ and the production of CO_2_. This Anaero-Pack system (MGC Inc., Japan) provided near-anaerobic conditions with an O_2_ concentration of <1% and a CO_2_ concentration of about 5% within 1h of incubation at 37°C. The cells were exposed to these conditions for 2 h, and then incubated again in glucose-containing DMEM at 37°C in 95% O_2_ and 5% CO_2_ (reperfusion) for 24 h.

### *In situ* cardiomyocytes apoptosis and quantitative analysis

A terminal deoxynucleotidyl transferase-mediated dUTP end labeling (TUNEL) assay was performed on cardiomyocytes that had been plated on flask-style glass slides. The *in situ* TUNEL assay was then performed in accordance with the manufacturer's protocol for cultured cells after fixing the cells in 10% neutral buffered formalin for 10 min at room temperature. Individual nuclei were visualized at a magnification of ×400 for quantitative analysis. An average of 400 to 500 nuclei was analyzed in random fields from each slide. The apoptotic index (percentage of apoptotic nuclei) was calculated as (apoptotic nuclei/total nuclei)×100%. Samples from at least 3 independent experiments were scored per group.

### RNA isolation and reverse transcription polymerase chain reaction (RT-PCR) of CaSR

Total RNA was extracted with Trizol reagent according to the manufacturer's instruction, and the concentration and purity of RNA were determined by measuring the absorbance at 260 nm, and 1 µg of total RNA was reversely transcribed into cDNA with 0.2 U/mL reverse transcriptase at 42°C for 50 min. The RT-PCR was performed by the ABI prism 7000 sequence detection system (Applied Biosystems, USA). The CaSR sense and antisense primers were 5′-TTCGGCATCAGCTTTGTG-3′ and 5′-TGAAGATGATTTCGTCTTCC-3′. A 230 bp band was obtained. The reaction conditions were: initial denaturation of 2 min at 94°C, 35 amplification cycles consisting of denaturation at 94°C for 20 s, annealing at 55°C for 40 s and elongation at 72°C for 40 s, with a final extension for 2 min at 72°C. The control PCR was performed with primers specific for the ubiquitously expressed endogenous β-actin gene, primers of sense and antisense were 5′-CGTTGACATCCGTAAAGAC-3′ and 5′-TGGAAGGTGGACAGTGAG-3′. A 201 bp band was obtained. PCR products were fractionated on 2% agarose gels and confirmed by sequencing (Chemi System Epichem 2, American UVP, USA). Levels of mRNA were normalized to β-actin, and expressed as relative mRNA levels compared with control.

### Western blot of caspase-3 and Bcl-2

Total neonatal rat myocyte proteins were prepared according to the manufacturer's instruction. At the end of the incubation period the medium was removed and the cells were washed twice with ice-cold PBS and incubated for 15 min in cool protein lysate containing the protease inhibitor phenylmethylsulfluoride (PMSF). Cells were then centrifuged at 1,400 *g* for 15 min at 4°C to remove the nuclei and undisrupted cells. The protein concentration in the supernatant was determined using a Bradford assay with BSA as standard. Total protein (20 µg) from samples were electrophoresed using standard 10% SDS-PAGE electrophoresis in Tris-Glycine buffer and blotted onto a nitrocellulose membrane in transferring buffer at 100 V for 1 h in a water-cooled transferring apparatus. The membranes were blocked in TBS-T buffer with 5% skimmed milk at 37°C for 1 h, and then incubated overnight at 4°C with anti-Bcl-2 (1:500)[21], anti-caspase-3 (1:500) and β-actin (1:10,000) antibodies. The membrane was then washed three times in TBS-T and incubated with anti-IgG antibody conjugated with alkaline phosphatase diluted 1:1,000 in TBS for 1 h at room temperature. Antibody-antigen complexes were detected using western Blue^®^ stabilized substrate for alkaline phosphatase. The protein bands were quantified by a Bio-Rad Chemi DOCTM EQ densitometer and Bio-Rad Quantity One software (Bio-Rad Laboratories, USA).

### Statistical analysis

All data were expressed as mean±SD. Differences among groups were analyzed by one-way analysis of variance (ANOVA), and Student-Newman-Keuls (SNK) method was used for multiple comparison. The *P*-values reported were two-sided, and values of *P* < 0.05 were considered statistically significant. All analyses were performed using SPSS software (Version 11.0, SPSS Inc., USA).

## RESULTS

### GdCl_3_ enhanced I/R-induced cardiomyocytes apoptosis and GdCl_3_ + NiCl_2_ + CdCl_2_ did not reverse this action

**Fig. 1:**
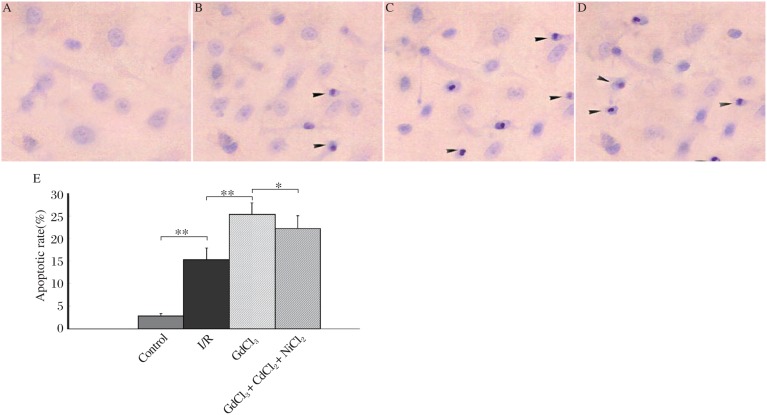
I/R- and GdCl_3_-induction of apoptosis in cultured neonatal rat cardiomyocytes. Arrowheads show cells with evidence of apoptosis, characterized by chromatin condensation (TUNEL,×400). A: normal cultured neonatal rat cardiomyocytes. B: I/R. C: GdCl_3_. D: GdCl_3_ + CdCl_2_ + NiCl_2_. E: apoptotic ratios of cardiomyocytes (*n* = 3, *P < 0.05, **P < 0.01).

TUNEL-positive cells contained characters of apoptosis, condensed chromatin and cellular shrinkage (***Fig. 1***). Cardiomyocytes exhibited significant apoptosis when exposed to I/R alone (*P* < 0.01 *vs* control). Pretreatment of GdCl3 further increased the apoptotic ratio (*P* < 0.01 *vs* I/R group). Although the apoptotic ratio in the GdCl_3_ + NiCl_2_ + CdCl_2_ group was slightly less than that in the GdCl_3_ group, the difference was not significant.

### I/R and GdCl_3_ increased CaSR mRNA expression in neonatal rat cardiomyocytes

**Fig. 2:**
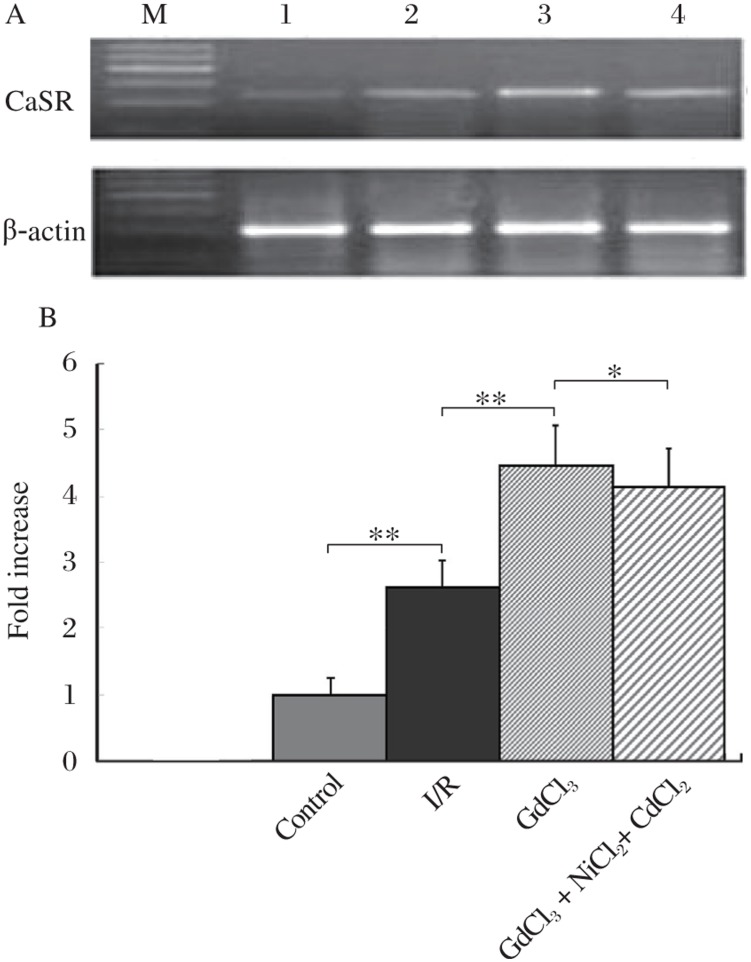
Detection of CaSR expression in cardiomyocytes by RT-PCR. A: RT-PCR results of CaSR. Lane M: Marker; Lane 1: control group; Lane 2: I/R group; Lane 3: GdCl_3_ group; Lane 4: GdCl_3_ + NiCl_2_ + CdCl_2_ group. B: Statistical analysis of RT-PCR results. I/R alone increased the CaSR expression compared to control. GdCl_3_ further increased CaSR expression. GdCl_3_ + NiCl_2_ + CdCl_2_ did not inhibit this action. (*n* = 3, *P < 0.05, **P < 0.01).

The expression of CaSR increased in I/R cardiomyocytes (*P* < 0.01 *vs* control group). Exposure of cells to medium containing GdCl_3_ further increased the CaSR expression (*P* < 0.01 *vs* I/R group). However, GdCl_3_ + NiCl_2_ + CdCl_2_ had no further effect (***Fig. 2***).

### GdCl_3_ down-regulated anti-apoptotic Bcl-2 and up-regulated pro-apoptotic caspase-3 cleavage expression

**Fig. 3:**
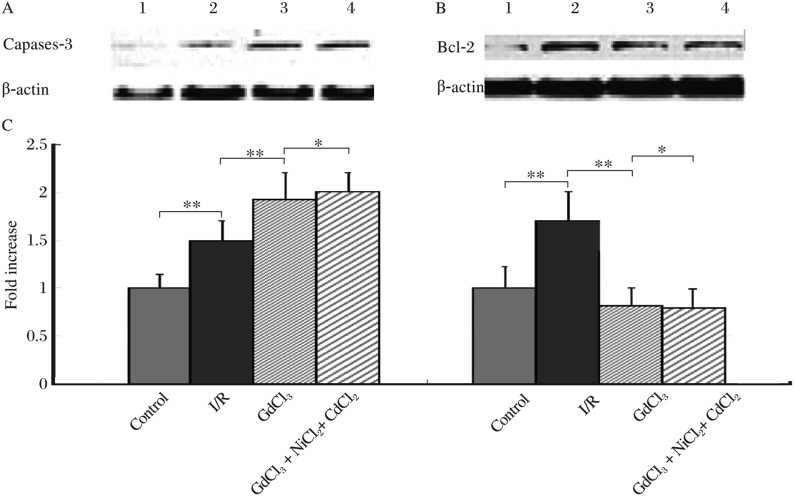
Detection of caspase-3 and Bcl-2 expression in cardiomyocytes by western blot. A: Detection of caspase-3 expression in cardiomyocytes by western blot. Lane 1: Control group; Lane 2: I/R group; Lane 3: GdCl_3_ group; Lane 4: GdCl_3_+NiCl_2_+CdCl_2_ group. B: Detection of Bcl-2 expression in cardiomyocytes by western blot. C: The statistical analysis of caspase-3 and Bcl-2 expression in different groups. The expression of caspase 3 was increased in all three ischemic groups, while the expression of Bcl-2 was only increased in the I/R group (*P < 0.05, **P < 0.01).

To identify the signaling pathway through which GdCl_3_, a specific activator of CaSR, induced apoptosis, we detected the expressions of anti-apoptotic Bcl-2 and pro-apoptotic caspase-3 by western blot. The expression of Bcl-2 was increased in myocytes treated with I/R, but GdCl_3_ significantly decreased the expression. Caspase-3 was significantly increased in the I/R group, and was further increased in the GdCl_3_ group, similar to the pattern seen in CaSR mRNA expression (***Fig. 3***).

The expression of Bcl-2 was increased in myocytes treated with I/R, but this effect was inhibited by GdCl_3_. On the other hand, both I/R and GdCl_3_ increased caspase-3 expression, and the increase in caspase-3 expression was significantly greater in the GdCl_3_ group than in the I/R group. Neither the L-type calcium channel blocker (CdCl_2_), nor the Na^+^/Ca^2+^ exchanger inhibitor (NiCl_2_) affected the GdCl_3_ action in I/R cardiomyocytes. The addition of NiCl_2_ and CdCl_2_ to GdCl_3_ had no significant effect on either Bcl-2 or caspase-3 expression.

## DISCUSSION

Previous studies showed that once the myocardium underwent severe ischemia, restoration of blood flow should be a prerequisite for myocardial salvage. However, there is a growing body of evidence that apoptosis of cardiomyocytes is one of the major contributors to myocardial infarction and to I/R injury[22]–[24]. As apoptosis occurs within 24 h and induces massive or submassive losses of myocytes, the susceptibility to cardiac dysfunction increases[25]. Therefore, if cardiomyocyte apoptosis could be inhibited, cardiac pathophysiologic changes and dysfunction due to myocardial infarction and I/R injury could be minimized[26]. Previous studies have shown that CaSR was expressed in rat neonatal and adult ventricular cardiomyocytes. However, little is known about the role of CaSR in I/R induced cardiac apoptosis. In the present study, we used GdCl_3_, a specific activator of CaSR, to investigate the role of CaSR in cardiomyocyte apoptosis-induced by I/R and to identify the signaling pathway involved. Our results revealed that apoptosis induced by simulated I/R was increased as the CaSR mRNA expression, and GdCl_3_, a specific activator of CaSR, further enhanced both the apoptosis and the CaSR mRNA expression. These findings suggest that CaSR activation is closely involved in cardiomyocyte apoptosis during I/R.

A stable concentration of calcium is important to the cell-cycle. Increased extracellular calcium commonly induces an increase of the intracellular concentration of calcium by three means[27]: voltage-gated calcium channels; Na^+^/Ca^2+^ exchanger; a receptor-mediated pathway. The use of Ca^2+^ as a ligand is not sufficient to prove the involvement of CaSR in calcium overload.

An intracellular calcium overload is regarded as a trigger for cardiomyocyte apoptosis during I/R injury. To determined whether involvement of CaSR in cardiomyocyte apoptosis during I/R is independent of calcium overload induced by the L-type calcium channel or Na^+^/Ca^2+^ exchanger, we detected CaSR changes after exposure of simulated I/R cardiomyocytes to GdCl_3_ and GdCl_3_ + NiCl_2_ (Na^+^/Ca^2+^ exchanger inhibitor) + CdCl_2_ (L-type calcium channel blocker). We found CaSR expression was further increased when GdCl_3_ was added. Neither the L-type calcium channel blocker nor the Na^+^/Ca^2+^ exchanger inhibitor blocked the GdCl_3_ action. This suggests that the apoptosis initiated by calcium overload was mainly induced by CaSR activation, but not via Na^+^/Ca^2+^ exchange or opening of L-type calcium channels. Massive intracellular calcium accumulation leads to the generation of a great deal of free radicals which results in mitochondrial deterioration, causing the release of apoptotic promotors and activation of the caspase cascade[28]. We thus regarded calcium overload triggered by CaSR activation as a likely cause of apoptosis in this simulated I/R model.

Our study showed the role of CaSR in induction of cardiomyocyte apoptosis involved in I/R was also investigated. Cardiomyocyte apoptosis detected by TUNEL staining was apparent in I/R, GdCl_3_ and GdCl_3_ + NiCl_2_ + CdCl_2_ groups. TUNEL positive cells in the GdCl_3_ and GdCl_3_ + NiCl_2_ + CdCl_2_ groups were significantly more than those in the I/R group. Meanwhile, apoptotic morphological changes of myocardiomyocytes were observed in all three ischemia groups (I/R, GdCl_3_ and GdCl_3_ + NiCl_2_ + CdCl_2_ groups), including nuclear chromatin marginalization, aggregation and condensation, and mitochondrion swelling. We believe that CaSR activation might increase intracellular calcium concentration and lead to calcium overload, resulting in apoptosis.

It has been demonstrated that apoptosis involves the intrinsic pathway (mitochondrial pathway) and extrinsic pathway (the receptor-mediated cell death pathway). The former is initiated by triggering cytosolic caspase-3 activation as a result of the formation of complex apoptosomes consisting of the cytochrome C/Apaf/caspase-9 when cells receive stress signals[29]. The latter is initiated by activation of initiator caspase-8 which can further stimulate the downstream effector caspase, such as caspase-3, when death receptors of the tumor necrosis factor receptor superfamily in cells are activated[30]. Caspase-3, a downstream caspase, is an important effector molecule in apoptosis[31]. In our study, caspase-3 expression was increased in I/R-induced apoptosis. The additional apoptosis seen in the GdCl_3_ group paralleled the increase in CaSR expression. So we hypothesized induction of cardiomyocyteapoptosis by CaSR is mediated by up-regulation of caspase-3.

Bcl-2 is a powerful inhibitor of apoptosis in response to a variety of cytotoxic stimuli[32]. Bcl-2 prevents the disruption of mitochondria, the subsequent release of cytochrome C and activation of the caspase cascade. Our study revealed that down-regulation of Bcl-2 expression occurred in the GdCl_3_-containing groups, accompanying the CaSR-induced apoptosis.

The modulation of apoptosis is clearly a complex process. All the signaling molecules involved are integrated into a single coherent network, and the quantities of pro-apoptotic and anti-apoptotic factors in the network determine the cell's final fate. When cells are responding to various kinds of cytotoxic stimuli, the quantities of pro-apoptotic and anti-apoptotic factors are changed. Thus the dynamic balance between pro-apoptotic and anti-apoptotic effects plays a critical role in determining the final cellular outcome after I/R injury. In our present study, apoptosis induced by CaSR activation up-regulated caspase-3 and in the GdCl_3_ groups down-regulated Bcl-2 expressions, suggesting the pro-apoptotic role of CaSR was dominant in I/R-induced cardiomyocytes.

In summary, these results suggest that CaSR activation is an important participant in I/R-induced apoptosis in cardiomyocytes from neonatal rats.
